# Unveiling the impact of simulated microgravity on HSV-1 infection, neuroinflammation, and endogenous retroviral activation in SH-SY5Y cells

**DOI:** 10.1007/s13365-025-01251-0

**Published:** 2025-03-20

**Authors:** Seyedesomaye Jasemi, Elena Rita Simula, Kawaguchi Yasushi, Leonardo Antonio Sechi

**Affiliations:** 1 Department of Biomedical Sciences, Division of Microbiology and Virology, University of Sassari, Sassari, Italy; 2 Division of Molecular Virology, Department of Microbiology and Immunology, The Institute of Medical Science, The University of Tokyo, Minato-ku, Tokyo, 108-8639 Japan; 3 Department of Infectious Disease Control, International Research Center for Infectious Diseases, The Institute of Medical Science, The University of Tokyo, Minato-ku, Tokyo, 108-8639 Japan; 4 Struttura Complessa Microbiologia e Virologia, Azienda Ospedaliera Universitaria Sassari, Sassari, Italy

**Keywords:** Microgravity, HSV-1, Herpes simplex virus 1, HERV, Immune response

## Abstract

Microgravity (µg) during spaceflight affects cellular and molecular functions of both human cells and microbial pathogens, influencing viral replication and the host immune system. This study aimed to investigate the effects of simulated µg on Herpes Simplex Virus-1 (HSV-1) replication, host pro-inflammatory cytokine, and human endogenous retrovirus (HERV) activation in human neuroblastoma SH-SY5Y cells. Our results show that µg has a negative impact on HSV-1 replication, leading to significantly reduced viral titers and lower expression levels of HSV-1 early genes (*ICP0*, *ICP4*, and *ICP27*) compared to 1 gravity (1 g) conditions. Interestingly, despite lower viral titers and HSV-1 gene expressions under µg condition, we observed higher levels of HERVs and pro-inflammatory cytokine gene expression. In addition, there was a significant correlation between HSV-1 immediate-early genes with HERVs and pro-inflammatory cytokine gene expression, with stronger correlations observed under µg conditions. Taken together, µg reduces HSV-1 replication and increases host pro-inflammatory and HERVs gene expression, which demands further investigation for human health protection in space.

## Introduction

Microgravity (µg), as forces less than 1 × 10^− 3^ g, is a key environmental factor during space exploration that affects the metabolism of both human cells and microbial pathogens (Juhl et al. 2021). It significantly influences several biological processes in human cells and microorganisms (Corydon et al. 2023; Huang et al. 2018; Marotta et al. 2024). Previous studies have demonstrated that exposure to microgravity can alter viral replication, modulate immune responses, and impact the regulation of host gene expression (Corydon et al. 2023; Fava et al. 2024; Long et al. 1999).

Herpes Simplex Virus type 1 (HSV-1), belonging to the *Alphaherpesvirinae* subfamily, is one of the most prevalent neurotropic pathogens that can infect the peripheral and central nervous systems (Roizman & Whitley 2007). After initial infection in epithelial cells, typically labial and oral lesions, HSV-1 establishes lifelong latency in trigeminal ganglia and reactivates periodically in response to stimuli such as stress, fever, and ultraviolet light exposure (Roizman et al. 2011). The HSV-1 reactivation can lead to a wide range of clinical outcomes, from mild cold sores to severe neurological disorders such as encephalitis (Arduino and Porter. 2008; Rechenchoski et al. 2017). The immune response to HSV-1 infection involves the activation of macrophages, natural killer (NK) cells, and cytotoxic T lymphocytes (CTLs), as well as the production of pro-inflammatory cytokines and chemokines, including interleukin-1β (IL-1β), interleukin-6 (IL-6), and tumor necrosis factor-alpha (TNF-α) (Amin et al. 2019). Emerging evidence also suggests that HSV-1 plays a role in neuroinflammation and neuronal dysfunction, processes that are closely associated with the pathogenesis of neurodegenerative disorders like Multiple Sclerosis (MS), Parkinson Disease (PD), and Alzheimer’s Disease (AD) (Bello-Morales et al. 2020, 2021).

Moreover, studies have demonstrated that herpesviruses may transcriptionally activate human endogenous retroviruses (HERVs) (Antony et al. 2011; Chen et al. 2019; Tao et al. 2017), and their combined activity highly increased cellular immune responses in both MS patients and healthy controls (Brudek et al. 2004). HERVs are a group of viral sequences integrated into the human genome over millions of years (Grandi and Tramontano. 2018; Johnson. 2019). HERVs, which constitute 5–8% of human genomic DNA, are largely silent under normal conditions. However, overexpression of HERVs has been associated with various proinflammatory diseases, including viral infection and neuroinflammatory disorders (Antony et al. 2011; Mao et al. 2021; Li et al. 2015).

Indeed, studies have consistently documented an increased frequency of latent viral reactivations, including herpesvirus, in astronauts during and after space missions (Mehta et al. 2017; Rooney et al. 2019). These reactivations are strongly related to immune system dysregulation caused by µg exposure. Although there is increasing evidence that immunological dysregulation in µg facilitates viral reactivation (Crucian et al. 2020), the specific impacts of µg on HSV-1 replication and its interactions with host cellular systems are yet inadequately understood. This study aimed to examine the effects of simulated µg on HSV-1 replication and its influence on host cellular responses, including the expression of inflammatory cytokines (*IL-1*, *IL-6*, *TNF-α*) and HERV genes (HERV-K *env*, HERV-W *env*, HERV-H *env*), in human neuroblastoma SH-SY5Y cells.

A random positioning machine (RPM) was used to create µg conditions by inducing rotation along three axes, simulating the space environment. SH-SY5Y neuroblastoma cells were selected as a recognized in vitro model for investigating neuronal activity, viral infections, and host-pathogen interactions. Our research provides new perspectives on the impact of microgravity on viral replication dynamics and host cellular responses, especially with pro-inflammatory cytokines and HERVs gene expression.

## Methods

### Virus titration, cell culture, infection, and simulated microgravity

The wild-type HSV-1 (HSV-1 F strain) was propagated in Vero cells, and then stored at − 80 °C until use. Before conducting experiments with the virus, the titre of the virus stock was determined by the plaque assay described previously (Shirahama et al. 2020).

Human neuroblastoma SH-SY5Y cells (CRL-2266, ATCC, Rockville, MD) were cultured in Dulbecco’s Modified Eagle’s Medium/F12 (DMEM/F12, Sigma-Aldrich, St. Louis, MI, USA), supplemented with 10% heat-inactivated foetal bovine serum (FBS) and a penicillin/streptomycin solution (100 U/ml and 100 µg/ml, respectively; both from Sigma-Aldrich). The cells were incubated at 37 °C in a humidified atmosphere containing 5% CO₂ and subcultured every 3–4 days.

For this experiment, 1 × 10^^5^ neuroblastoma SH-SY5Y cells were seeded in T12.5 flasks (n: 34) with DMEM/F12 containing 10% FBS, 100 U/ml penicillin, and 100 µg/ml streptomycin were incubated at 37 °C in a humidified incubator with 5% CO2 until they reached 90% confluence.

To optimize the multiplicity of infection (MOI), we performed preliminary experiments using different MOIs (MOI = 0.1, 0.5, and 1) to evaluate the appropriate viral load for detecting significant differences in HSV-1 replication under simulated microgravity (µg) versus normal gravity (1 g). Our results indicated that MOI = 1 provided a detectable level of viral replication while avoiding excessive cytopathic effects that could confound our analysis.

Sixteen of the flask cells were infected with HSV-1 at a MOI of 1. The virus was allowed to adsorb to cells for 2 h at 37 °C in rotating conditions, after which the viral inoculum was removed and replaced with fresh medium. Other sixteen flasks change supernatant with fresh medium. Eight flasks of HSV-1-infected SH-SY5Y cells and eight flasks of non-infected SH-SY5Y cells were incubated under µg using a random positioning machine (RPM, Fokker Space, Netherlands) at the laboratory of the Department of Biomedical Sciences, University of Sassari, Sardinia, Italy. The same number of flasks was incubated under normal gravity (1 g) conditions at 37 °C in a humidified atmosphere containing 5% CO2. The cells were harvested at different time points: 3 h (*n* = 2 infected and 2 non-infected SH-SY5Y flasks under microgravity and, the same for 1 g conditions), 6 h (*n* = 2 infected and 2 non-infected SH-SY5Y flasks under microgravity and, the same for 1 g conditions), and 24 h (*n* = 2 infected and 2 non-infected SH-SY5Y flasks under microgravity and, the same for 1 g condition.

### Trypan blue staining and viable cell calculation

The cell suspension was collected, 50 µL of which was put into an Eppendorf tube and then mixed well with 50 µL of 0.2% trypan blue dye, followed by dropping into the cell counting plate. After standing for 2 min, the numbers of living (unstained) and dead cells (stained blue) were counted, and the percentage of living cells was calculated as the number of unstained cells/the number of total cells × 100%.

### Vitus Titration

At each time point, the cells were collected, and Plaque assay method was performed as described previously (Shirahama et al. 2020). Briefly, infected SH-SY5Y cells were collected on a duplicate set at different times post-infection and mixed with 500 medium and then subjected to three freeze-thaw cycles so that the virus was released into the medium. The cell lysates from infected cultures were serially diluted and added to Vero cell monolayers in 6-well plates for 1 h to allow virus attachment and entry into the cells. Then, the supernatant was aspirated, and 2 ml of medium freshly supplemented with pooled human IgG (Merck) was added and incubated at 37 °C and 5% CO_2_ for 3 days. For plaque counting, the cells were fixed with 10% formaldehyde for 20 min and stained with 0.1% (w/v) crystal violet staining solution for 10 min. HSV-1 titre was calculated through plaque counting and expressed as PFU/ml.

$$\:pfu/ml=\frac{number\:of\:plaques\:per\:well}{dilution*infection\:volume\:\left(0.5\:ml\right)}$$

### RNA extraction, reverse transcription, and Real-Time PCR

To investigate the expression of viral genes and cytokine-related genes in SH-SY5Y cells following incubation in 1 g and RPM-simulated µg conditions at various time points, we conducted quantitative real-time PCR (qRT-PCR) analyses. Total RNA was isolated using the Qiagen RNeasy Mini Kit in accordance with the manufacturer’s instructions. cDNA was synthesized from 2 µg of total RNA using the Thermo Fisher Scientific RevertAid RT Kit. The expression levels of viral genes, including ICP0, ICP4, and ICP27; human endogenous retroviruses (HERVs), including HERV-K env, HERV-W env, and HERV-H env, and inflammatory cytokines (IL-1, IL-6, and TNF-α), were quantified using SYBR Green Master Mix (Applied Biosystems) and specific primers (Table 1) (Li W; Ruberto et al. 2022; Wei et al. 2021; Zhang et al. 2005). mRNA expression was normalized to the amount of GAPDH and actin rRNA expression. The relative amount of each gene expression was calculated using the comparative cycle threshold (fold change).

**Table 1:** The primers used in this study

Target gene	Sequence 5′ to 3′ (Forward)	Sequence 5′ to 3′ (Reverse)
IL1B	F: GCACGATGCACCTGTACGAT	R: AGACATCACCAAGCTTTTTTGCT
IL6	F: CCAGGAGCCCAGCTATGAAC	R: GAGCAGCCCCAGGGAGAA
TNF-α	F: CAGAGGGAAGAGTTCCCCAG	R: CCTTGGTCTGGTAGGAGACG
GAPDH	F: CAAGGAGTAAGACCCCTGGAC	R: TCTACATGGCAACTGTGAGGAG
HERV-H env	F: CCCATATTTGGACCTCTCAC	R: TGTGTAGTTGGGCTTTGGAG
HERV-W env	F: CCTATTTAATACCACCCTCACTG	R: AGTTGTTCCATTGTTCAGGT
HERV-K env	F: GCTGTCTCTTCGGAGCTGTT	R: CTGAGGCAATTGCAGGAGTT
ICP0	F: TTACGTGAACAAGACTATCACGGG	R: TCCATGTCCAGGATGGGC
ICP4	F: GGCCTGCTTCCGGATCTC	R: GGTGATGAAGGAGCTGCTGT T
ICP27	F: GTCTGGCGGACATTAAGGAC A	R: TGGCCAGAATGACAAACAC G
Actin	F: ACGAGGCCCAGAGCAAAGA G	R: TCTCCATGTCGTCCCAGTTG

### Statistical analysis

#### Statistical analysis

Statistical analysis was conducted using GraphPad Prism 8.0 software (GraphPad, San Diego, CA, USA). Three independent experiments were conducted for each step, each including three technical replicates. Data distribution was analyzed using the Shapiro–Wilk test and Kolmogorov-Smirnov test. Gene expression differences between microgravity (µg) and normal gravity (1 g) conditions were analyzed using an unpaired two-sided Student’s t-test. If the data were normally distributed, Pearson’s correlation test was employed to evaluate the correlation between gene expression. Non-parametric data were analyzed using Spearman’s rank correlation test. A *p*-value of less than 0.05 was considered statistically significant.

## Results

### Impact of simulated microgravity on HSV-1 replication and -related genes expression

**Fig. 1:**
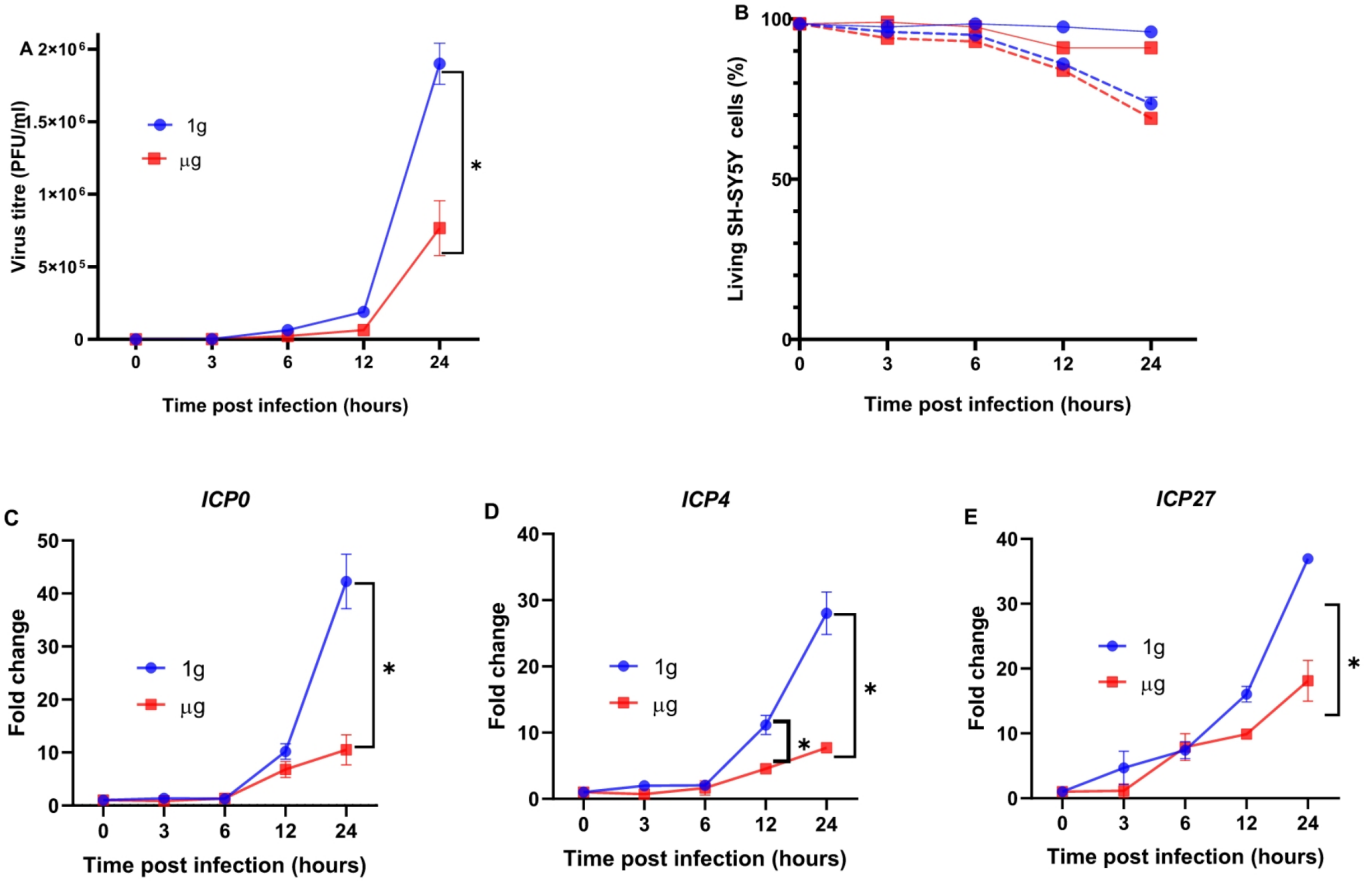
(**A**) Virus titers (PFU/mL) of HSV-1 at different time points (3, 6, 12, and 24 h) in SH-SY5Y cells under 1 g (blue line) and µg (red line) conditions. (**B**) Percentage of living SH-SY5Y cells under 1 g (blue line) and µg (red line) conditions, with living HSV-1-infected SH-SY5Y cells under 1 g (blue dashed line) and living HSV-1-infected SH-SY5Y cells under µg (red dashed line) conditions. Expression patterns of (**C**) *ICP0*, (**D**) *ICP4*, and (**E**) *ICP27* at different time points after incubation under 1 g (blue line) and µg (red line) conditions. Data are presented as the average fold change ± SD. Statistical significance is indicated by an asterisk (*), with *p* < 0.05

The viral titre was measured at multiple time points post-infection (3, 6, 12, and 24 h) (Fig. 1A) to assess the effect of simulated microgravity on HSV-1 replication. Our results showed that at earlier time points (3, 6, and 12 h), no significant differences were observed in the measured viral titers between 1 g and µg conditions. However, after 12 h, a substantial increase in viral replication was observed under 1 g compared to simulated µg (*p* < 0.001) (Fig. 1A).

Furthermore, since HSV-1 replication is stimulated by the expression of its early genes, we analysed the expression levels of three key HSV-1 immediate-early genes, *ICP0*, *ICP4*, and *ICP27*, at different time points post-infection (3, 6, 12, and 24 h) under 1 g and simulated µg conditions to confirm the viral titer assay results (Fig. 1C, D, and E). At early time points (3 and 6 h), no significant differences in gene expression were observed between 1 g and µg conditions. However, by 24 h, we found a marked increase in the gene’s expression under 1 g compared to µg conditions (*p* < 0.001) (Fig. 1C, D, and E). The strong agreement between qRT-PCR and plaque assay results further confirmed the inhibitory effect of simulated microgravity on HSV-1 replication.

In addition, the lowest percentage of SH-SY5Y cell viability was observed in virus-infected SH-SY5Y cells under µg conditions, although this difference was not statistically significant compared to virus-infected SH-SY5Y cells under 1 g conditions (Fig. 1B). A similar trend was also observed in non-infected SH-SY5Y cells between µg and 1 g conditions (Fig. 1B).

### Inflammatory cytokines and HERVs gene expression were variable under Μg condition

**Fig. 2:**
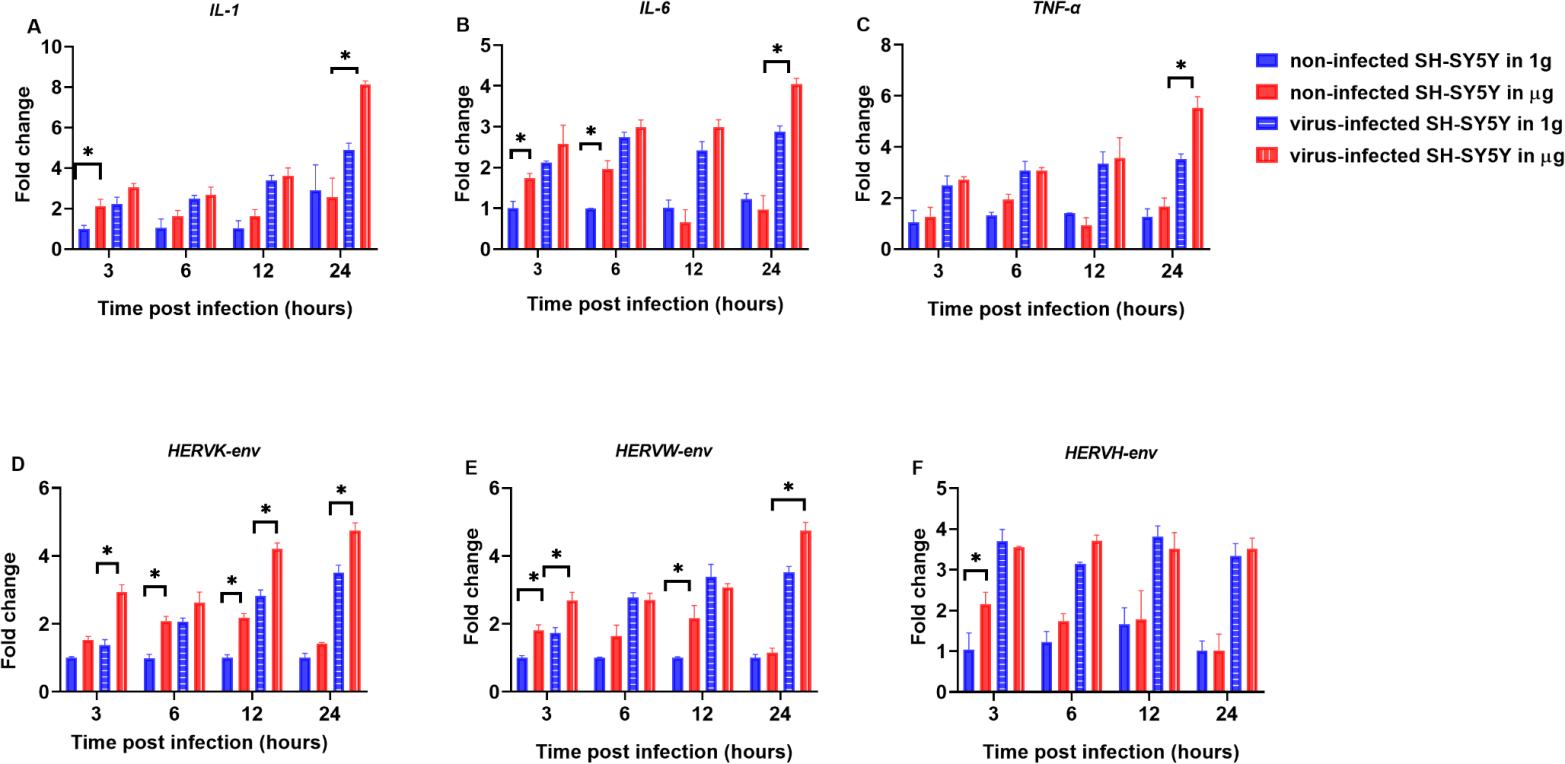
Expression patterns of immune-related genes and HERV genes in SH-SY5Y cells at different time points (3, 6, 12 and 24 h) under various conditions. The blue bar represents SH-SY5Y cells under 1 g conditions, while the red bar represents SH-SY5Y cells under µg conditions. The blue striped bar denotes SH-SY5Y cells infected with HSV-1 under 1 g conditions (1 g*), and the red striped bar indicates SH-SY5Y cells infected with HSV-1 under µg conditions (µg*). Bars display the average fold change ± SD of gene expression levels. *Indicates statistical significance (*p* < 0.05). Expression patterns of (**A**) *IL-1*, (**B**) *IL-6*, (**C**) *TNF-α*, (**D**) HERV-K *env*, (**E**) HERV-W *env* and, (**F**) HERV-H *env*

We measured the expression levels of pro-inflammatory cytokines (*IL-1*, *IL-6*, and *TNF-α*) and HERV genes (HERV-K *env*, HERV-W *env*, and HERV-H *env*) in non-infected and virus-infected SH-SY5Y cells under 1 g and simulated µg conditions at various time points (Fig. 2).

In non-virus-infected SH-SY5Y cells, the expression of *IL-1* and *IL-6* genes was significantly increased when exposed to short-term µg (3 h, 6 h), compared to 1 g conditions (Fig. 2A and B). Then, the expression levels of both *IL-1* and *IL-6* genes in cells under µg condition returned to levels comparable to those observed under 1 g conditions. The *TNF-α* gene expression did not change when SH-SY5Y cells were exposed to µg (Fig. 2C). In parallel, in non-infected SH-SY5Y cells subjected to µg, HERVs *env* gene expression levels changed significantly over time (Fig. 2D, E, and F). HERV-K *env*, HERV-W *env*, and HERV-H *env* exhibited significantly higher expression levels at primary hours of incubation (at 6 h for HERV-K *env*, 3 h for HERV-W *env*, and HERV-H *env*) under µg conditions compared to the 1 g condition (*p* < 0.05). However, by 12 h for HERV-K *env*, 24 h for HERV-W *env*, and 6 h for HERV- H *env*, the expression levels in µg aligned with those observed under 1 g condition (Fig. 2D, and F).

Additionally, in virus-infected SH-SY5Y cells, a steady increase in the expression levels of both pro-inflammatory cytokines and HERV genes was observed over time under both 1 g and µg conditions (Fig. 2D, E, and F). However, this upregulation was more pronounced under µg. Notably, the expression of *IL-1*, *IL-6*, and *TNF-α* genes in virus-infected cells was significantly higher at 24 h under µg compared to 1 g conditions (*p* < 0.05) (Fig. 2A, B, and C). Furthermore, HERV-K *env* and HERV-W *env* showed significant upregulation as early as 3 h of incubation in µg, with this trend persisting and remaining significantly elevated at 24 h compared to 1 g conditions.

Although an increase in the HERV-H *env* expression levels was observed under µg, the differences were not statistically significant when compared to 1 g conditions.

### Correlation analysis of gene expression in 1 g and µg conditions

**Fig. 3:**
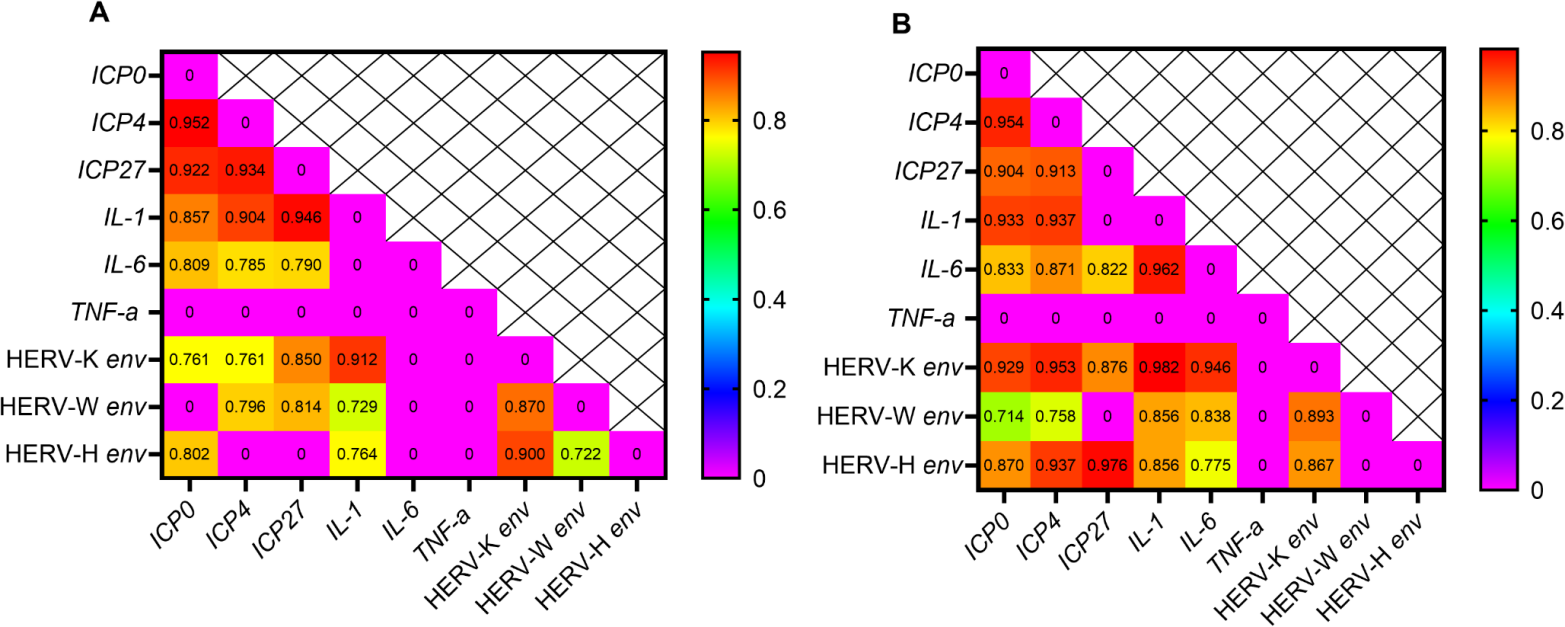
Heat map of genes expression fold change. (**A**) Gene expression fold changes under 1 g condition, (**B**) Gene expression fold changes under µg condition

Finally, we evaluated the correlation between viral expression (HERVs and HSV-1) and cytokine gene expression under 1 g and µg conditions. Our analysis showed a similar correlation pattern in both 1 g and µg conditions. Figure 3 shows a statistically significant correlation (*p* < 0.05).

There was a significant correlation between the expression of HSV-1 and HERV genes. Under 1 g conditions, a positive correlation was observed between *ICP0*-HERV-K *env* (*r* = 0.761, *p* = 0.036) and *ICP0*-HERV-H *env* (*r* = 0.802, *p* = 0.021), whereas, under µg conditions, correlations were observed among *ICP0* -HERV-K *env* (*r* = 0.929, *p* = 0.000), *ICP0* -HERV-W *env* (*r* = 0.714, *p* = 0.04), *ICP0* -HERV-H *env* (*r* = 0.870, *p* = 0.003), *ICP4* -HERV-K *env* (*r* = 0.953, *p* = 0.000), *ICP4* -HERV-W *env* (*r* = 0.758, *p* = 0.02), *ICP4* -HERV-H *env* (*r* = 0.937, *p* = 0.000), *ICP27*-HERV-K *env* (*r* = 0.870, *p* = 0.004), and *ICP27*-HERV-H *env* (*r* = 0.976, *p* < 0.0001).

Moreover, in 1 g conditions, there was a significant correlation between HERVs and *IL-1*, specifically involving HERV-K *env* -*IL-1* (*r* = 0.912, *p* = 0.001), HERV-W *env* -*IL-1* (*r* = 0.729, *p* = 0.03), and HERV-H *env* - *IL-1* (*r* = 0.764, *p* = 0.02), whereas under microgravity conditions, this correlation was apparent between HERVs with both *IL-1* and *IL-6*. These correlations encompassed HERV-K *env* - *IL-1* (*r* = 0.982, *p* < 0.0001), HERV-W *env* - *IL-1* (*r* = 0.856, *p* = 0.006), HERV-H *env* - *IL-1* (*r* = 0.856, *p* = 0.006), HERV-K *env* - *IL-1* (*r* = 0.946, *p* = 0.0004), HERV-W *env* - *IL-1* (*r* = 0.838, *p* = 0.0093), and HERV-H *env*- *IL-1* (*r* = 0.775, *p* = 0.023).

## Discussion

This study investigated the effects of simulated µg on the replication of HSV-1 and host-cell responses, focusing on pro-inflammatory cytokines and endogenous retrovirus (HERVs) expression in SH-SY5Y cells.

For the first time, our findings showed that simulated µg conditions significantly reduced HSV-1 replication in SH-SY5Y cells compared to 1 g. This result was evident from the reduced virus titers, and decreased expression levels of critical HSV-1 immediate-early genes (*ICP0*,* ICP4*, and *ICP27*) under µg conditions.

The replication of HSV-1 is entirely dependent on the energy resources and molecular mechanism of the host cell (Copeland Anna et al. 2009). Thus, any detected reduction in replication could result from the effect of microgravity on the host cell, the virus, or a combination of both.

Studies indicate that microgravity inhibits cell proliferation through decreased expression of essential cell cycle regulators and cytoskeletal proteins, alongside a reduction in ATP production (Marotta et al. 2024). The alterations in host cell functionality under µg conditions may substantially influence HSV-1 replication efficiency. Previous studies on SH-SY5Y cells under µg indicate that elevated nitric oxide (NO) production contributes to increased oxidative stress (Bi et al. 2009; Wang and Good. 2001), which is closely associated with reduced ATP levels in host cells, potentially impairing the energy supply required for efficient HSV-1 replication. In our study, we observed that non-infected, viable cells under microgravity conditions exhibited a slight, albeit non-significant, decrease in number compared to those under 1 g. While this difference was not statistically significant, it indicates that microgravity may affect non-infected SHSY-5Y cells, which could affect their overall viability.

Furthermore, research indicates that µg exerts both suppressive and reactivating effects on various members of the herpesvirus family as well as on unrelated viruses (Long et al. 1999; Mishchenko et al. 2004). Long et al. (1999) conducted a study that demonstrated a 7- to 25-fold decrease in the expression of early and late Epstein-Barr virus (EBV) genes, such as ZEBRA and VCA, in lymphoblastoid cells grown in rotating-wall vessels (Long et al. 1999). In addition, a study showed that simulated µg conditions reduced the viral reproduction rate and its spread in wheat plants infected with the wheat streak mosaic virus (WSMV) (Mishchenko et al. 2004). Contrary to the suppressive effects observed in simulated microgravity, studies on astronauts during real spaceflight reported increased expression of both latent and lytic EBV genes and productive replication observed in long-duration spaceflight conditions in peripheral blood (Stowe et al. 2011). Data from space missions indicate a shift from latency to active replication of herpesvirus in B lymphocytes, driven by immunosuppression and elevated stress during spaceflight (Mehta et al. 2017; Rooney et al. 2019). These conditions likely differ significantly from those in simulated microgravity environments, where other risk factors such as radiation, circadian disruption, and psychological factors are absent.

In this study, we also measured the expression of pro-inflammatory cytokines and endogenous retroviruses (HERVs) in infected and non-infected SH-SY5Y cells under simulated µg and 1 g conditions. In non-infected SH-SY5Y cells, we observed an upregulation in the expression of cytokines and HERV genes under simulated µg conditions during the first 12 h of incubation in a clinostat. However, by 24 h, the expression levels decreased and were the same as 1 g condition. There are rare studies that have been performed on HERV gene expression under µg conditions (Jasemi et al. 2025; Wu et al. 2024). Recent study has shown reactivation of HERVs in human immune cells after 25 h of incubation under simulated µg (Wu et al. 2024). In addition, we observed a significant increase in the expression of pro-inflammatory and HERV genes in infected SH-SY5Y cells compared to non-infected SH-SY5Y cells, especially when the SH-SY5Y cells were exposed to µg condition. Although in non-infected cells, we observed a significant upregulation of HERV and inflammatory cytokine genes under µg conditions compared to those under 1 g conditions. This result aligns with our previous study, which demonstrated the upregulation of ERV genes and inflammatory cytokine genes (IL-1, IL-6, TNF-α) under simulated µg conditions in various cell lines (Jasemi et al. 2025). Similarly, a study using monocytic leukemia THP-1 cells found that µg significantly increased both mRNA and protein levels of TNF-α, IL-6, and IL-1β, especially when combined with lipopolysaccharide (LPS) stimulation (Zhang et al. 2018). This finding highlights the possibility of an exacerbated inflammatory response in the context of spaceflight and associated microgravity exposure.

Various studies have shown that herpesviruses can trigger a significant inflammatory response and HERV activation, especially in neuronal disorders (Brudek et al. 2004; Ruprecht et al. 2006). For instance, HERV-W gag and env proteins are induced by herpes simplex virus type 1 (HSV-1) in neuronal and brain endothelial cells *in* vitro (Ruprecht et al. 2006). In addition, combinations of the endogenous retrovirus HERV-H and herpes virus antigens resulted in highly increased cellular immune responses among both the MS patients and healthy subjects (Brudek et al. 2004). However, despite lower HSV-1 gene expression and viral titers in µg compared to 1 g conditions, we observed higher HERVs and pro-inflammatory cytokine gene expression under µg conditions, which probably shows direct dysregulation of the immune system and HERVs under µg conditions. Although lower HSV-1 gene expression, and viral titers in µg allow the host immune system to respond effectively, that need for future studies to fully understand these dynamics.

However, this study has several limitations. First, using a random positioning machine (RPM) to simulate microgravity may not fully replicate the complex effects of actual spaceflight conditions. Additionally, the short-term exposure to simulated microgravity (up to 24 h) limits insights into its long-term effects on HSV-1 replication and host cell responses. Moreover, protein expression analyses of cytokines and HERVs were not performed, which could have further validated the qPCR results.

## Conclusion

This study demonstrated that microgravity reduces HSV-1 replication while increasing inflammatory responses and HERV activation, two mechanisms associated with inflammatory disorders. These findings may have implications for spaceflight-associated health risks, where astronauts are exposed to microgravity and may experience viral reactivation.

## Data Availability

No datasets were generated or analysed during the current study.
